# Prognostic Factors for Conversion to Arthroplasty after Hip Arthroscopy. Review of the Literature

**DOI:** 10.1055/s-0041-1741512

**Published:** 2021-12-30

**Authors:** Roberto Seijas, David Barastegui, Ferran Montaña, Marta Rius, Xavier Cuscó, Ramón Cugat

**Affiliations:** 1 Instituto Cugat Hospital Fundació García Cugat Quiron, Barcelona, Spain; 2 Fundació García Cugat, Garcia, Cugat; 3 Medicine Department, Universitat Internacional de Catalunya, Barcelona, Spain; 4 Mutualitat Catalana de Futbolistas (FCF)—Real Federación Española de Futbol, Madrid, Spain

**Keywords:** hip arthroscopy, prognostic, prognostic factor, total hip replacement, conversion, arthroplasty

## Abstract

Arthroscopic techniques in the treatment of femoroacetabular impingement have experienced an exponential increase over recent years for both diagnosis and treatment. The main risks with treatment are poor clinical outcomes and the conversion to prosthesis. Better knowledge and understanding of the various risk factors leading to prosthesis will improve patient selection for arthroscopic treatments rendering better results. The published papers that have been selected are related to series of hip arthroscopies with risk factors that lead to total hip arthroplasty (THA), in the PubMed database, without a time limit, number of patients, or follow-up time. We selected over 302 papers, 19 papers that show risk factors for conversion to THA. The main risk factors found were femoral chondropathy grade III/IV (relative risk 58.1–12 times increased), acetabular (20–2.96 times), an articular space <2 mm (39–4.26 times), age (14.6–1.06 times), Tönnis 2 in radiographic studies (7.73–3.1 times), obesity (5.6–2.3 times), and osteoarthritis (4.6–2.4 times). There are several risk factors which in an isolated way, highly increase the risk of THA. Some of them have a clear relationship (chondropathy, reduced joint space, Tönnis 2, and osteoarthritis). Based on the review we observed that the elements that are most associated with a conversion to THA after a hip arthroscopy are a high degree of femoral and acetabular chondropathy, a reduced joint space below 2 mm, older age, Tönnis 2, obesity, and hip osteoarthritis.

Osteoarthritis of the hip has a significant clinical affectation in today's population producing pain and functional alterations. 1 2 It is estimated that between 10 and 19 million inhabitants of the United States 2 are affected and management involves an expenditure of approximately 24 billion USD in Australia each year. 2

Given this impact, early treatment is aimed at improving the quality of life of these patients and consequently reducing a very high health expenditure. 2 3

Different studies have linked hip osteoarthritis with femoroacetabular impingement and postulate that treatment can alter at least the symptomatic impact for these patients, although long-term studies are needed to see if it modifies the course of the disease. 2 4 5 6

Hip arthroscopy has proven effective in solving coxofemoral joint problems, especially in femoroacetabular impingement. 7 The use of this technique has seen exponential growth in the last decade. In the United States, between 2004 and 2009 it increased 365%, between 2006 and 2010 600%, 8 and between 2007 and 2011 250%, 9 while in the United Kingdom between 2002 and 2013 the increase was 727%. 10 This increase has been observed in those under 30, with an increase of 355% (between 2007 and 2011), but also in those over 60 (200% in the same period). 9 11 It is estimated that the annual increase for this technique could be as much as 15%. 2 12

There are several justifications for this increase, such as: advances and improvements in the technical aspect, improvements in the indications, 9 13 14 15 better results in young patients and athletes, 16 greater exposure of the population to a greater number of hours of sport, 9 and the greater demand by the population to maintain their standard of living as well as that of sports. 9

Another factor to take into account is the increase in the spectrum of indications, such as femoroacetabular impingement, labral lesions, and cartilaginous lesions, with the latter being the most frequent to be treated by this technique. 17 18 19 20

Hip arthroscopy has demonstrated its efficacy both in the short 13 21 and long term, 20 22 23 with regards to clinical improvement, with good and excellent results in pathologies such as FAI and labral lesions, 16 24 25 26 27 28 29 without degeneration in studies published at longer follow-up. 7

But this increase in indications also leads to an increase in the number of revision surgeries, including conversions to total hip arthroplasty (THA). 30 31 In fact, the main reason for re-operating after an arthroscopy is to perform a conversion to THA. 26 A percentage of these conversions to THA (and hip arthroscopy failure) should be considered attributable to an incorrect preoperative diagnosis or poor patient selection. 32 33

The preoperative detection of factors that can provide us with information regarding the viability of our surgical procedure 32 33 is vital to increase the success rate of this technique.

This study is based on the systematic review of published studies with the objective of identifying which prognostic factors are related to a higher rate of conversion to THA after hip arthroscopy.

## Material and Methods

A bibliographic search is performed using the keywords: *Hip Arthroscopy, Hip Replacement Arthroplasty, Total Hip Replacement, Prognosis, Risk Factors* , in different combinations in the PubMed database in April 2019.

The inclusion criteria were those articles that included risk factors leading to THA, with no follow-up limit or limit on the number of participants. The exclusion factors were articles that did not clearly expose the risk factors, opinion articles or editorials, isolated cases or systematic reviews that did not provide their own series.

**Fig. 1:**
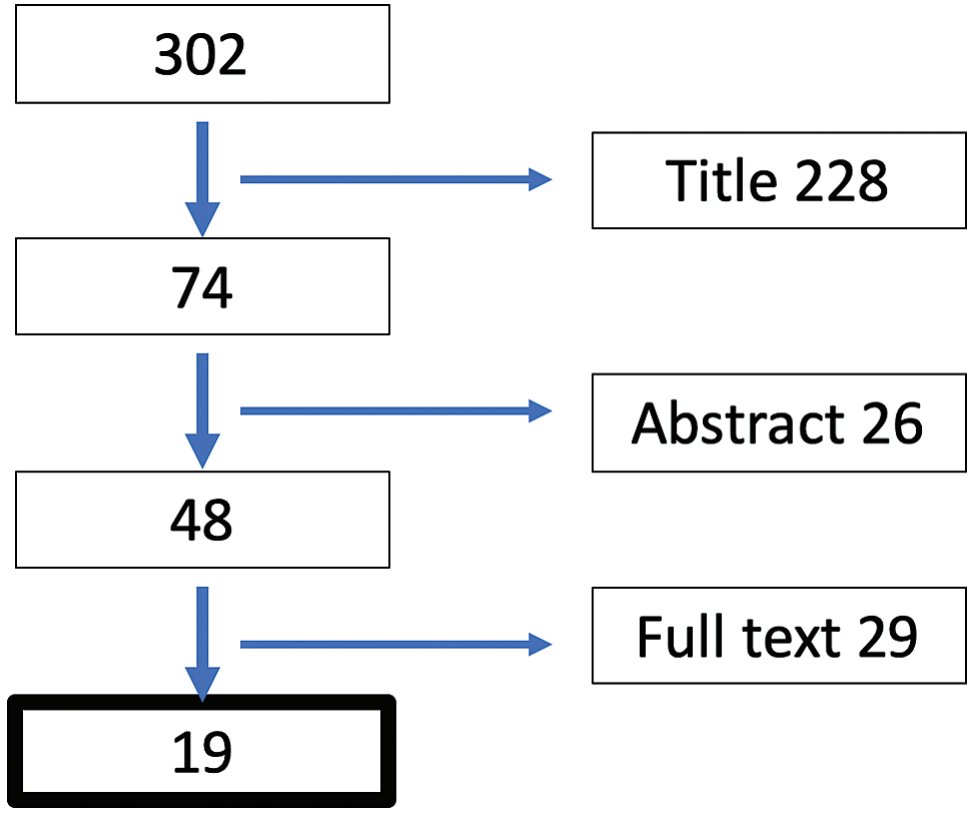
Flowchart of the study, where the studies were eliminated according to the title, the summary, and after reading the full text.

Initially, 302 articles were selected from which 74 were selected after reviewing the titles, eliminating isolated clinical cases, opinion articles, and articles unrelated to our question. Twenty-six articles were withdrawn from the review because they did not adhere to the topic defined when reviewing the summary. The 48 remaining articles were reviewed in full text format and once again eliminating the articles that did not fit our study, with the final sample of 19 articles that were selected to perform this review ( Fig. 1 ).

## Results

The articles included in this study present case series with a considerably varied sample, with series from 30 cases up to 8,827. This is due to the larger series being obtained from database reviews, while the shorter series are series performed by a specific group of surgeons reviewing their own cases.

Risk factors showed significant differences in each study and a statistical calculation of the relative risk was performed. In some of the studies this calculation was not performed and therefore is not reflected in the table. Table 1 shows the data collected from the 19 articles.

**Table 1:** Summary of the selected articles, with the risk factors to THA after an arthroscopy and the relative risk they presented in each work

Author (Ref)	Year	*N*	Risk factor	RR
Kaldau et al (34)	2018	84	Age >40 y Cartilage lesion	– –
Kester et al (35) c	2018	3,957	>60 y Osteoarthritis Tobacco Obesity Female Less skilled surgeon	3.4 2.4 1.9 5.6 1.8 1.9
McCarthy et al (12)	2018	989	Osteoarthritis Age >50 y Prior arthroscopy	4.6 3.8 2.6
Perets et al (45)	2018	94	Tönnis >0 Femoral Outerbridge >2	3.1 12
Haefeli et al (50)	2017	52	LCE angle >33 degrees Acetabular index <3 degrees	4.6 –
Menge et al (40)	2017	145	Age Space <2 mm Acetabular microfractures	3.06 4.26 2.86
Redmond et al (41)	2017	792	Age mHHS low Femoral anteversion Revison surgery Outerbridge II Outerbridge III Outerbridge IV Acetabuloplasty Lack of femoroplasty	1.06 0.98 0.97 2.4 2.23 2.17 2.96 1.83 0.62
Bedard et al (11) c	2016	1,728	Age >50 Chondroplasty Osteoarthritis	3.18 3.5 3.8
Chandrasekaran et al (44)	2016	935	Tönnis 2	7.73
Herrmann et al (30)	2016	99	Osteoarthritis Joint space	– b – b
Schairer et al (39) c	2016	7,351	Age >60 (although >40 risk increases) Obesity Osteoarthritis Centre volume >10/y	14.33 2.43 2.3 0.76
Malviya et al (36) c	2015	6,395	Age >50 Female sex	4.65 1.68
Redmond et al (38)	2015	30	Age >60 Preop mHHS <50 Preop VAS >7	−2.6 2.3
Sing et al (9) c	2015	8,227	Age >50 Osteoarthritis	– –
Skendzel et al (6)	2014	466	Space <2 mm Alpha >55 degrees	10,8 a 2.1 a
Philippon et al (42)	2013	96	Space <2 mm Tönnis two-thirds	12 4.8
Philippon et al (43)	2012	153	Space <2 mm Pre mHHS <60 p	14.6 3.2
McCarthy et al (37)	2011	106	Age >40 y Outerbridge III/IV acetabular Outerbridge III/IV femoral	3.6 20 58.1
Philippon et al (3)	2009	122	Space <2 mm	39
Total	–	31,821		

Abbreviations: LCE, lateral center edge; mHHS, modified Harris Hip score; RR, relative risk; THA, total hip arthroplasty; VAS, visual analogue score.

a Risk of having grade III/IV Outerbridge in surgery.

b Significantly higher risk in the THA group, without calculation of relative risk.

c 86.9% of the cases belong to five papers with very long series.

Risk factors are also not always defined equally ( Table 2 ). Age is considered in some works as a dichotomous variable, above or below a figure, which can be 40, 50, or 60 years. 9 11 12 34 35 36 37 38 39 Some studies find that the risk is progressive and does not depend on a limit value, but it is an increasing risk as one grows older. 39 40 41

**Table 2:** Risk factors ordered from highest to lowest, according to the literature review

Risk factors	Relative risk
Chondropathy grade III/IV femoral	58.1(37) 12(45)
Articular space <2 mm	39(3) 14.6(43) 12(42) 10.8(6) 4.26(40)
Chondropathy grade III/IV acetabular	20(37) 2.96(41)
Age	14.6(39) 4.65(36) 3.8(12) 3.6(37) 3.4(35) 3.18(11) 3.06(40) 1.06(41)
Tönnis 2	7.73(44) 4.43(42) 3.1(45)
Obesity	5.6(35) 2.3(39)
Osteoarthritis	4.6(12) 3.8(11) 2.4(35)
LCE angle greater than 33 degrees	4.6(50)
Need to perform chondroplasty	3.5(11)
mHHS value prior to surgery less than 60 points	3.2(43) 2.6(38)
Acetabular microfractures needed	2.86(40)
Previous surgery	2.6(12) 2.4(41)
VAS preop greater than 7	2.3(38)
*α* angle greater than 55 degrees	2.1(6)
Lacking surgical experience	1.9(35)
Female sex	1.8(35) 1.68(36)
Tobacco	1.9(35)
Need for acetabuloplasty	1.8(41)

Abbreviations: LCE, lateral center edge; mHHS, modified Harris Hip score; VAS, visual analogue score.

Other variables, such as osteoarthritis, may be defined by radiological criteria or not included in the work as such, because the data that is linked to the operated patient is analyzed. 9 11 12 30 35 39 Some of the works take the reduced articular space into account, 3 6 40 42 43 some based on the Tönnis classification, 42 44 45 and some works are based on the state of the cartilage, according to the Outerbridge classification. 34 37 41 45

## Discussion

With this article the authors attempt to review the prognostic risk factors after a systematic review of the literature and the main published works.

Age is a prognostic factor. 9 12 36 37 38 39 There is a higher conversion rate in older patients, 11 36 with figures from 5.6 to 38.8% during the 1st year and >80% in the long term. 6 9 22 36 37 Age as an isolated factor has been associated with an increased risk of conversion to THA. 9 37 39 40 Patients older than 50 years have been associated with rates of 13 to 20% conversion in 1.6 to 2 years, 9 12 42 although some studies have shown good results with that age although with shorter series (20 good results on 22 patients). 46 Longer term series, at 8 and 10 years of follow-up, show rates of 37 to 38.8% in the group >50 years. 36 37

Age over 60 is considered as an increased risk of THA from 3.4 47 times to 14.33. 39 The study by Kaldau et al shows that 87% of patients who converted to THA were older than 40, while those who kept their hip preserved, only 46% were over 40. 34 In this same study, being 40 years old represents a 29% risk of leading to THA in a period of less than 8 years. 34 Along these same lines, other studies have similar findings. 6 40 48 The study by Domb et al presents better results, with a conversion rate to THA of 17.3% in those aged over 50. 49 The study by Sing et al 9 shows 16% of those over 50 with follow-up at 2 years, or Schairer et al 39 with 12.4% THA also with more than 2 years of follow-up, although the conversion rate was lowest in patients aged younger than 40 years, with a relative risk of 5.48, above 50 years places it at 8.97, and above 60 years at 14.33. 39 Some studies present as little as 4% with follow-up rates of 7 to 9 years. 50 However, another study by Domb and colleagues 38 in which the subgroup above 60 years old is analyzed, findings show a 30% rate of prosthesis in just over a year, in the same way the knee studies also showed similar results. 51

The female gender has been detected as a factor of greater risk in some works, although not with homogeneous results in all the reviewed works, 37 45 presenting a risk of 1.68 36 to 1.8 35 compared with males.

An articular space of <2 mm has been associated with joint pain 40 52 53 and worse prognosis 3 6 and hip arthroscopy has only shown a temporary improvement in these patients. 7 Patients with a previous joint space of <2 mm have shown 43% and 86 conversion rates to THA in less than 3 and 5 years, as opposed to 10 and 16% who have followed the same path if they presented an articular space >2 mm in preoperative radiography. 6 42 43 The presence of a reduced coxofemoral joint space on preoperative radiographs is associated with a 10.8-fold increased risk of presenting high-grade chondral lesions (Outerbridge III/IV) in arthroscopy, 6 and an increase in the risk of conversion to THA of 39 times 3 (especially if the population is over 50 years old, where the risk rises 12 times 42 ). The presence of an *α* angle >55 degrees increases the risk by 2.1 times. 6 45

An acetabular coverage angle (Tonnis or Acetabular index) of less than 3 degrees 50 is an increased risk. A Wiberg angle (lateral center angle) >33 degrees increases the risk by 4.6 times, 50 although another study finds that the risk is other way around, with a higher risk at lower angles. 45 Hip osteoarthritis is one of the risk factors to consider. 11 12 30 37 54 55 Cases with advanced osteoarthritis do not improve clinically with this procedure, although the initial phases may have a clinical benefit. 7 39 56 57 The presence of degenerative changes classified as Tönnis two-thirds increases the risk of THA by 4.8 times. 42 Another study warns of the risk even for patients with Tönnis 1, especially in those over 50 where risk of THA is increased by 3.1 times. 45 The Domb group observed a risk in Tönnis 2 patients to THA in 2 years of 7.73 with respect to Tönnis 0 and 4.36 with respect to Tönnis 1. 44

The progression of clinical pain and degenerative joint disease can lead to conversion to THA, which according to published studies is around 16 to 37% at 6 to 11 years follow-up. 7 34 37 40 45 54 Different authors have conversion rates of 2.9 to 22.8% between 2 and 3 years after arthroscopy, 27 30 39 54 with 63% joint survival rates at 10 years of follow-up. 37 40 When we analyze how many of the patients who required THA presented osteoarthritis, we found that almost all of them had osteoarthritis of the hip. 9 The presence of level 4 ALAD lesions, (associated with more than 80% of THA in patients in less than 2 years) may even suggest the conversion during an arthroscopy in the same surgical procedure. 38

In addition, conversions are usually early with a staggering 36% of conversions occurring during the first 6 months, 60% in the first year and 100% of cases during the first 4 years. 9 11 36 41 54 Patient data must be analyzed to assess whether the chosen surgery can or should be offered. 58

Although the exact causes, 22 37 58 are unknown, one of the factors to consider is both acetabular and femoral cartilage injury. 34 A degree of Outerbridge injury, 37 45 54 59 lesions of the chondrolabral complex (ALAD), 38 45 or the need for cartilage treatment have shown a higher conversion rate 11 37 41 in both the acetabulum and femoral head. 34 40 45 Grade III/IV acetabular or femoral lesions have been described as factors of poor prognosis, increasing the possibility of ending up as a THA in less than 10 years with an increase of 20 and 58.1 times respectively, with femoral head injuries posing a greater threat than those of the acetabulum. 37 45

The described factors have an obvious relationship. Chondropathy, loss of joint space, osteoarthritis, and a Tönnis 2 are expressions of joint degeneration. That is why the different studies that evaluate the different points of evolution show different degrees of risk. In a perspective study they can be taken into account in a global way.

There are also other risk factors to consider in light of this review. Tobacco use is related to an increased risk of 1.9. 35 Obesity (body mass index >30) increases the risk from 2.43 to 5.6 times. 35 39 60 61 62

Both the surgeon's experience (<40 hip arthroscopies annually) and centers with a lower hip arthroscopy volume, increase the risk of THA conversion, 35 (1.9 times in low-volume surgeons). In centers where at least 10 hip arthroscopies are performed per year, there is a reduced risk of THA conversion of 0.76. 39

In addition, the articles have shown how over time, rates of prosthesis are reduced, without necessarily reducing patient age. Schairer et al 39 presented rates of prosthesis of 14.3% in 2005 while in 2010 they were 10.3% with significant differences. It is very likely that both experience and skill in the diagnosis and detection of the factors of poor prognosis have contributed to this decline, a point that will most likely see further improvement in the coming years.

Inflammatory arthritis is also an isolated risk factor for conversion to THA. 63 64

The history of a previous hip arthroscopy, that is to say, hip re-arthroscopy increases the risk of conversion to THA by 2.6 times. 12

Regarding subjective assessment questionnaires such as visual analogue scale (VAS) pain level or modified Harris Hip score (mHHS), they have also shown their prognostic value. A preoperative value of mHHS below 50 points increases the risk of conversion to THA by 2.6 times, and below 60 points in a population over 50 years increases by 3.2 times. 43 If a VAS greater than 7 the risk is 2.3 times higher. 38

The combination of some of those factors is really surprising. The combination of age and cartilage lesions may have a high prognostic component. Age below 40 years and (I/II) Outerbridge injuries are related to a 10% rate of prosthesis at 10 years follow-up, while >40 years and Outerbridge III/IV at 10 years has a rate of prosthesis of 99%. 37

The present study has several limitations to take into account. This is a review, which, despite being systematic, may contain bias in its search and combination of terms. To reduce this limitation we have followed the steps that can be observed in the flowchart.

Second, the articles reviewed present a very different number of patients. Series of highly expert groups in this type of surgery have been collected with reviews of global databases that mix the results of different groups with differences in their level of experience. That is why the results may differ. The work of expert teams is normally associated with the great experience of the leader of that group, while data reviews could offer a more homogeneous photograph of a larger segment of surgeons.

Third, we have detected that many of the variables could be superimposable. A reduced space and Tonnis 2, grade 3 to 4 chondropathy and osteoarthritis could be synonymous terms and they may share a high percentage of similarities in the eyes of most observers.

## Conclusion

Based on the review we observed that the elements that are most associated with a conversion to THA after a hip arthroscopy are high grades of femoral and acetabular chondropathy, reduced joint space below 2 mm, older age, Tönnis 2, obesity, and hip osteoarthritis.
